# Prediction of Bacterial Contamination Outbursts in Water Wells through Sparse Coding

**DOI:** 10.1038/s41598-017-00830-4

**Published:** 2017-04-11

**Authors:** Levi Frolich, Dalit Vaizel-Ohayon, Barak Fishbain

**Affiliations:** 1 Technion Enviromatics Lab (TechEL), Faculty of Civil and Environmental Engineering, Technion – Israeli Institute of Technology, Haifa, 3200003 Israel; 2 Mekorot – National Water Analysis Lab, Israel National Water Company, Eshkol, P.O.B 610, 1710502 Israel

## Abstract

Maintaining water quality is critical for any water distribution company. One of the major concerns in water quality assurance, is bacterial contamination in water sources. To date, bacteria growth models cannot predict with sufficient accuracy when a bacteria outburst will occur in a water well. This is partly due to the natural sparsity of the bacteria count time series, which hinders the observation of deviations from normal behavior. This precludes the application of mathematical models nor statistical quality control methods for the detection of high bacteria counts before contamination occurs. As a result, currently a future outbreak prediction is a subjective process. This research developed a new cost-effective method that capitalizes on the sparsity of the bacteria count time series. The presented method first transforms the data into its spectral representation, where it is no longer sparse. Capitalizing on the spectral representation the dimensions of the problem are reduced. Machine learning methods are then applied on the reduced representations for predicting bacteria outbursts from the bacterial counts history of a well. The results show that these tools can be implemented by the water quality engineering community to create objective, more robust, quality control techniques to ensure safer water distribution.

## Introduction

The necessity to preserve high water quality is incumbent on all water utilities. One of the concerns, a water company must address, is contamination as a result of bacterial outbursts in water sources. The main tool for coping with these outbursts is early detection through bacteria counts, which are done on a regular-basis.

Bacteria counts time series are governed by two major forces: (i) bacteria growth, and (ii) sampling rate. The natural behavior of bacteria growth occurs in exponential spurts as opposed to linear generation, and is highly unpredictable because of the myriad phenomena that affect bacteria growth^1^. The natural microbial quality of the water tends to have low bacteria concentrations, leading to many zeroes in the measurement’s time series at unpredictable indexes, classifying them as **sparse**. This results in a non-Gaussian distribution of the counts^1^. These counts’ atypical distributions prevent a smooth application of mathematical models and therefore make the monitoring and prediction of bacteria outbreaks difficult. Given the difficulty of utilizing mathematical models, the *normative state* of a well is currently determined at the discretion of an appointed engineer. The engineer uses his or her own subjective reasoning and in essence makes educated guesses. This process is neither objective nor efficient. Consequently, a new objective methodological approach is necessary to better model bacteria outbreaks in water wells.

Several mathematical models have been suggested for describing bacteria growth. Peleg & Horowitz^2^ propose a model which is based on daily measurements of fecal coliforms acquired in a water reservoir over *three years*. While they show that, in principle, it is possible to model the bacteria growth after a statistical distribution and estimate its parameters, even with such an extensive data set, the authors admit that they require much longer and more complete time series to refine their model to the point that it would be able to predict the frequency of future outbursts. Hadas *et al*.^3^ present a model based on the Laplace distribution density, using water samples taken every 2–4 weeks from the sea of Galilee (i.e., Lake Kinneret), where indicator organisms (*E. coli*, enterococci and fecal coliforms) were then analyzed. They calculate the distribution parameters by fitting the empirical data to the distribution by applying a transformation of order *n* ^*1/3*^, the method of moments and the maximum likelihood method. While the fit to the distribution is satisfactory, the authors state that their model cannot predict when an outburst will occur, but only when the frequencies of counts exceed a given level. Schnabel *et al*.^4^ conducted a study looking into how seasonality and flow regimes affect the bacterial growth distribution. One of their conclusions is that the fecal coliform measurements do not adapt to any known distribution. Hence, all the above models were found to be “site specific”, thus, the fact that the distribution characterized the measurement data, only attests that in this particular case the model works. This finding does not mean the calculated parameters or distribution has transferable applicability to other cases. Hence, these types of models only provide for long terms population means and would not be able to predict sudden outbursts.

System dynamics^5^ aims at representing a complex system through the circular, interlocking, and time-delayed relationships among its components. Dynamic models have been shown to model adequately many biological complex environments such as epidemiological spread^6^ and ecological processes^7–9^. In the context of modeling bacteria growth, Dolgonsov *et al*.^10^ presented this kind of model; however, they concede that it is simple as often there is a dearth of data, which impedes the evaluation of the various parameters. This is due to the irregular sampling regime mandated by the standards.

Another approach to water quality monitoring is the application of statistical quality control (SQC) methods, developed for the control of industrial processes, to water quality monitoring either through Shewhart^11, 12^ or CUSUM^12, 13^ charts. To this end, Smeti *et al*.^11^ tested the possibility of using Shewhart charts to monitor the turbidity, aluminum and residual chlorine in water storages. MacNally and Hart^13^ examined the use of CUSUM charts for the control of water storages, specifically phosphorous counts. Zhou *et al*.^12^ looked at the combination of CUSUM and Shewhart charts for monitoring environmental impacts of karst terrenes. While these studies found that Shewhart and CUSUM may offer an effective way to monitor water quality, the required assumptions preclude their use for bacteria counts. This is because, for both Shewhart and CUSUM, the parameter distribution must approach normality and the counts must be independent. These two assumptions tupically do not hold for bacteria counts. Furthermore, the variance of the observed parameter must be known or well estimated and must also remain constant through time. CUSUM charts are particularly sensitive to strong seasonal variation; and Shewhart charts can be used for fast detection of large shifts in the process; however, they cannot detect small shifts. Thus, both approaches would not be useful for bacterial counts.

Sandle^14^ developed a method to ameliorate the inherent deficiencies in microbial time series so that they can be used in SQC methods. He suggests transforming the data by the power of *n* ^*1/2*^, which worked in his specific data set. But this does not guarantee that it will be appropriate for other data sets. In that research, after applying Sandle’s transformation, the data did not tend to any recognizable distribution. This is in line with Hadas *et al*.^3^ who used a transformation of order *n* ^*1/3*^ for their data set, showing that there is no universal transformation and every case must be evaluated individually.

The use of machine learning techniques to model bacteria growth or event detection has become increasingly more prevalent. The use of such techniques as artificial neural networks for bacteria growth prediction has been explored by a number of researchers^15–17^. The models use other indicators to predict bacteria growth (as opposed to the bacteria counts themselves) in surface water and therefore would require a change in the sampling regulations to demand that water suppliers sample a broader range of indicators.

Modaresi and Araghinejad^18^ examined different machine learning methods for the classification of water quality. They found that the Support Vector Machine (SVM) algorithm^19^ can be calibrated and validated to determine the existence of contaminates in water. However, they did not apply their methods on bacteria, and they focused their research on classification as opposed to prediction of new events before they occur. Oliker and Ostfeld^20^ applied SVM and a minimum volume ellipsoid (MVE)^21^ algorithms for contamination event detection in a water system. While their methods showed that SVM and MVE can be effective in separating between normal and out of control operation, their approach was not applied specifically to bacteria growth.

It is evident that the prevalent water quality control methods are deficient. The current bacteria growth models cannot be applied universally and the SQC methods are difficult to apply to the unique characteristics of bacteria count time series. Currently, the most promising area of research is machine learning algorithms; however, to date, no algorithm has been proposed for the prediction of bacteria outbursts in water wells. The objective of this research was to develop a new method of predicating bacterial contamination that takes into account the unique nature and sparseness of bacterial counts time series. This method set out to provide the basis for a tool that the global water quality engineering community will implement.

This research hypothesized that viewing the bacteria time series as a signal and capitalizing on its natural sparseness would allow for better prediction of bacteria growth. The flow of logic is as follows:

1. In their current state, bacteria time series are difficult to analyze practically due to their sparseness.
2. Spectral methods ameliorate the difficulties presented by the sparseness of the time series, allowing for a better prediction of an outburst based on a small subset of the spectral coefficients, thus reducing the dimensions of the problem^22–24^.

The justification for the coefficient or dimension reduction is twofold: First, any deviation in bacteria counts from the norm would manifest itself vividly, as the change in counts is now spread over a small number of coefficients; second, it alleviates the curse of multidimensionality^22–24^, improving the classification to normal and high-risk states.

The reminder of this paper is organized as follows. The results presents the performance the suggested scheme using MVE^21^ and SVM^19^ algorithms and their comparison to Artificial Neural Network classification (ANN). The discussion summarizes the results  and concludes the paper. The  methods section, describes the data used in this study, and the dimension reduction and classification mechanisms.

## Results

### Dimension Reduction

This research viewed the bacteria time series as a signal that could be analyzed through use of spectral analysis methods. This approach allows for reducing the dimension of the problem, so a future outburst can be better predicted using only a small number of spectral coefficients^22–24^. Two mechanisms for dimension reduction are presented: signal energy and sparse coding. The energy of a signal is preserved over all its unitary transforms^25^ and can be used as a measure of activity in the well. The motivation for using signal’s energy is that it describes the signal with a single number, ultimately reducing its dimensions. Sparse coding aims at finding a transform that represents signals in a compact form.

#### Energy

The energy algorithm was run over four active bacteria time series acquired in four different active water wells in Israel - Rosh Hayin 4, Rosh Hayin 5, Rosh Hayin 7 and Rosh Hayin 8. The sampling information is given in Table 1.

**Table 1:** Rosh Hayin wells sampling information.

	Rosh Hayin 4	Rosh Hayin 5	Rosh Hayin 7	Rosh Hayin 8
Start Date	4/4/2001	21/2/2001	11/3/2003	21/2/2001
End Date	26/10/2011	26/1/2011	14/11/2011	26/12/2011
Number of samples	212	268	255	318
Mean Time Between Samples [days]	19	15	12	12
Max Count [CFU/100 ml]	120	120	120	120

**Figure 1:**
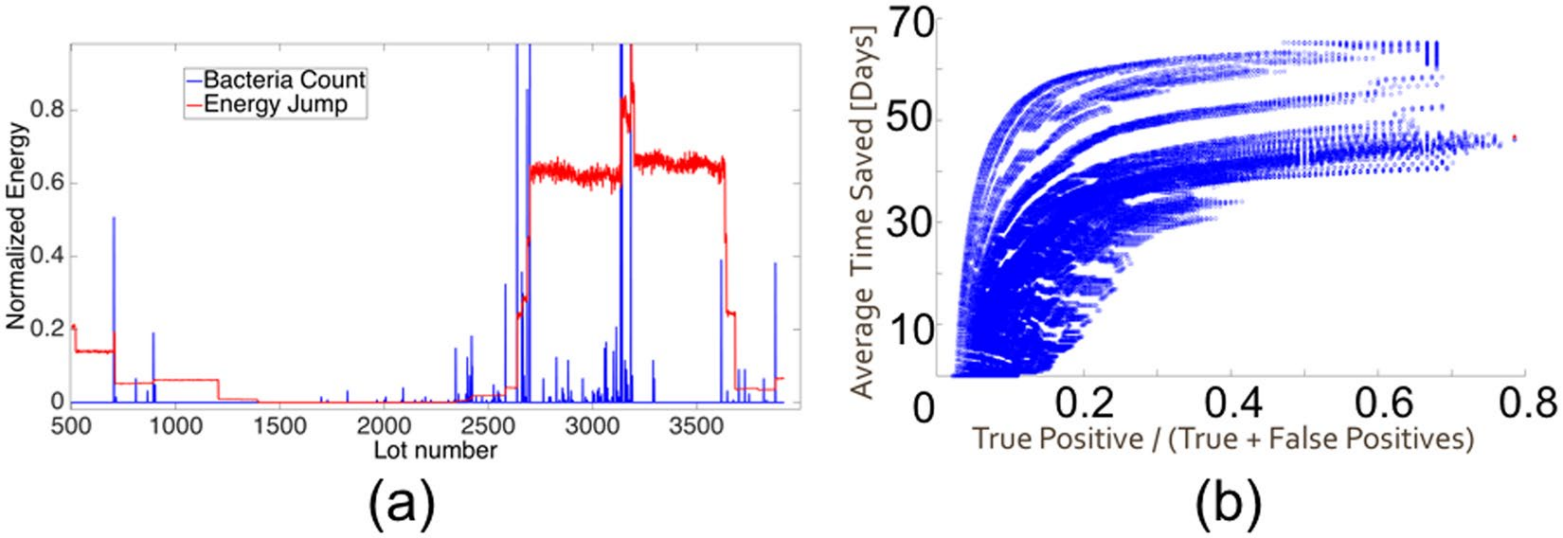
Energy Analysis. (**a**) Rosh Hayin 4 bacteria normalized count (blue) versus the corresponding normalized Energy jump (red); (**b**) Pareto Frontier, i.e., average time saved vs. true positives/(Total number of positives) for Rosh Hayin 7 well.

Figure 1a depicts the energy and the bacteria time series, normalized (i.e., divided by its maximum value). In order to run the energy algorithm, the energy threshold by which to throw an alarm and the window size, by which the energy change is computed, had to be determined. This was determined by calculating the Pareto frontier^26^, where the average time saved versus the false positive percentage were used as the parameters. An example is given in Fig. 1b.

An important conclusion drawn from this analysis was that each well had its own optimal window size and threshold. Thus, it is impossible to generalize either a threshold or a window size. This is to be expected as the literature has shown that wells are generally idiosyncratic. The results from the optimization are found in Table 2.

**Table 2:** Threshold and Window Size.

Well	Threshold	Window Size [days]
Rosh Hayin 4	1244	326
Rosh Hayin 5	1396	321
Rosh Hayin 7	1332	301
Rosh Hayin 8	544	420

The energy moving window algorithm was run on each well, using its unique threshold and window size. The results are summarized in Table 3.

**Table 3:** Energy Algortihm Results.

Well	True positive rate	Average time saved [days]
Rosh Hayin 4	1.00	49
Rosh Hayin 5	0.53	31
Rosh Hayin 7	0.79	47
Rosh Hayin 8	0.44	114

#### Dimension Reduction

**Figure 2:**
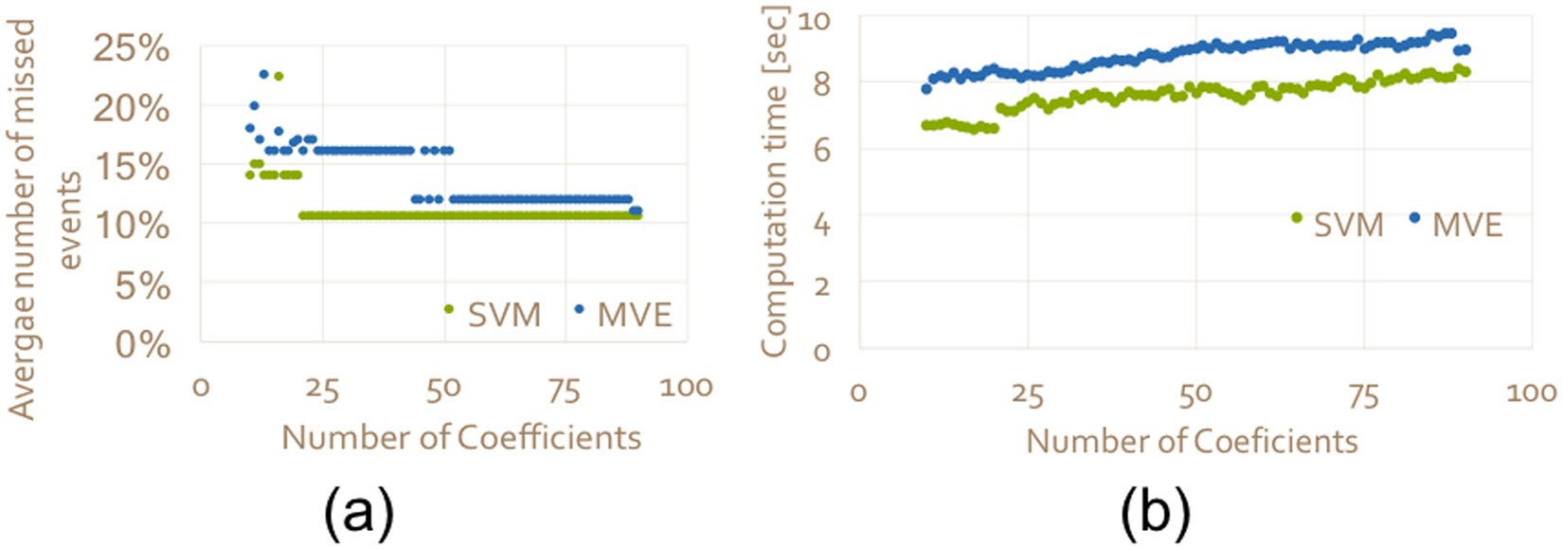
The effect of the number of coefficients on the results. (**a**) Average percentage of missed events for SVM (green) and MVE (blue) computed for Rosh Hayin 4, Rosh Hayin 5, Rosh Hayin 7 and Rosh Hayin 8 water wells vs. the number of coefficients used in the prediction window; and (**b**) the computation time (in seconds) for SVM (green) and MVE (blue) as a function of the number of coefficients used.

As explained in the introduction, the concept here is based on the notion that given the bacteria time series are sparse, a small number of frequency coefficients express changes in counts and deviations from the norm in a vivid way. To test this, both SVM and MVE were run with a different number of coefficients for the aforementioned water wells, Rosh Hayin 4, Rosh Hayin 5, Rosh Hayin 7 and Rosh Hayin 8. Both computation time and the number of missed events were analyzed, the results of which are shown in Fig. 2. On average, as the number of coefficients increases the computation time increases. When the dataset becomes significantly large, this might limit the use of the algorithm. The minimum number of coefficients necessary is 10. Furthermore, at a certain point (for SVM it is 20 and for MVE around 43 coefficients) adding more coefficients does not improve the results. This implies that the sparsity of the time series is exploited and most of the information can be gleaned from far fewer coefficients. Therefore, it was decided to use 30 coefficients for both SVM and MVE.

### Classification Results

The SVM and MVE algorithms were run on the same four active bacteria counts time series: Rosh Hayin 4, Rosh Hayin 5, Rosh Hayin 7 and Rosh Hayin 8. One measure used to test the success of the prediction was to determine how many days prior to the event the algorithm predicted the bacteria outburst. This was simply calculated by taking the lot number where the event occurred minus the lot number where the first prediction occurred within the 90-days window. The results were then averaged so that each well had an “average time saved” metric.

To better assess the performance of the suggested method, the results of the proposed scheme are compared against an Artificial Neural Network (ANN) outburst prediction mechanism^27^. ANN and its variations are common tools for modeling and predicting bacteria growth and may serve as a valid comparison^28–32^. ANN, similarly to SVM and MVE, consists of training and validation phases. It is important to note that all three algorithms were executed after dimension reduction has been applied. The same historical training window, for each well, that facilitated the calibration of the SVM and MVE, was used for training and validating the neural network, applying the Levenberg-Marquardt algorithm^33, 34^. The ANN takes two parameters as user input: *f* - The number of hidden neurons, thus, the number of processing layers; and *d*, the delay, i.e., how many coefficients are used to infer the next coefficient:

1 $$y(t)=f(y(t-1),y(t-2),\cdots ,y(t-d))$$

Both *f* and *d* were found through an exhaustive search for the minimum normalized mean squared error (nMSE) between the signal generated by the trained network and the training data. These parameters are detailed in Table 4.

**Table 4:** Optimal historical window length (History); number of hidden layers (*f*); and delay (*d*) for classification between normal and high-risk states.

Well	History	*f*	*d*
Rosh Ayain 4	2254	24	19
Rosh Ayain 5	1500	6	13
Rosh Ayain 7	1239	13	10
Rosh Ayain 8	2254	14	17

Table 4 reiterates the notion that each well presents a different behavior. For each well, the prediction of future outbursts, using the above historical window size (for SVM, MVE and ANN) and the optimal number of hidden layers and delay (for ANN only), is carried out. The results are summarized in Table 5.

## Discussion

As can be seen in Table 5, most of the events were predicted using both SVM and MVE and both outperform ANN. The algorithms often correctly identified contaminated and non-contaminated windows (even those non-contaminated windows that contained bacteria counts that were non-zero). The average time predicted for each well was the highest for MVE with comparable results for SVM. Similarly to the detection rates, ANN presents shorter saved times.

**Table 5:** MVE SVM and ANN Results. Percentage of False Negatives, False Positives and Time saved on average, i.e., how many days in advance on average an alarm was raised before the actual outburst.

	False Negatives			False Positives			Time Saved		
	MVE	SVM	ANN	MVE	SVM	ANN	MVE	SVM	ANN
Rosh Ayain 4	**8**	**8**	43	**0**	**0**	60	**87**	60	67
Rosh Ayain 5	**4**	12	41	1	**0**	22	**75**	22	47
Rosh Ayain 7	**3**	5	43	3	**0**	44	**83**	44	55
Rosh Ayain 8	50	**17**	88	**22**	44	20	**86**	20	3

ANN algorithm comes short in predicting future outbursts compared to MVE and SVM, having said that, it was proved to be efficient in describing bacterial growth^28–32^. This discrepancy may be attributed to the fact that in previous studies the modeling focused on a single growth process rather than fluctuating time series. Therefore, we conclude that in general MVE and SVM with spectral representation of the signal can be used as a predictive algorithm for bacteria growth. The following discussion looks at several particular snippets to highlight important phenomena and the differences between the MVE and SVM algorithms.

**Figure 3:**
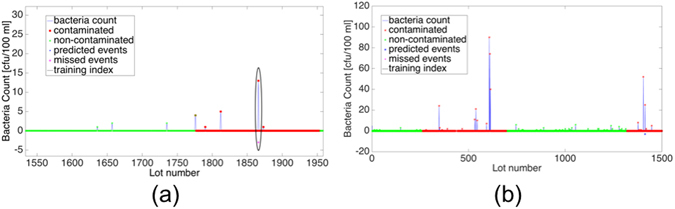
Rosh Hayin 5 SVM Analysis. (**a**) A snippet from Rosh Hayin 5 counts time series (April 1^st^, 2005 – June, 30^th^, 2006), where one can identify a classification error; (**b**) presents the historical training window (February 21^st^, 2001–June 30^th^, 2005).

Figure 3a depicts a section from the Rosh Hayin 5 SVM prediction time series, which represents the time frame between April 1^st^, 2005 and June 30^th^, 2006. This section shows a steady increase in bacteria growth, and therefore the algorithm should have predicted the event (highlighted by a circle). Figure 3b is the training historical section of the same time series, which corresponds to February 21^st^, 2001 through March 31^st^, 2005. The contaminated windows of the training section (Figure b) do not conform cleanly to the typical bacteria growth model of exponential growth. Rather, there are a few peeks amidst several lower values. Therefore, the SVM training would have difficulty in identifying the pattern of bacteria growth exhibited in a. This highlights the sensitivity of the algorithm to training. The cleaner the training is, the better the outcome. One way to achieve a better training is to have more history to train on. As the time series progresses the algorithm can continually update by retraining on the new time stamps.

Most of the missed events (False negatives) were a result of no preceding information. This is expected as the algorithm only predicts an event, if there is bacteria growth leading up to the event.

**Figure 4:**
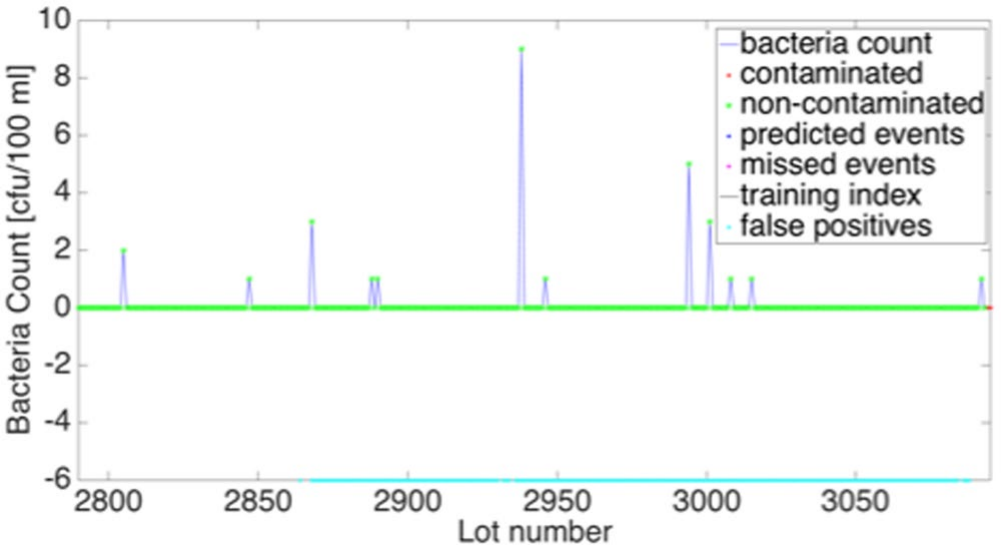
SVM prediction – Snippet from Rosh Hayin 8 time series Oct. 2008–Aug. 2009.

Rosh Hayin 8 had the highest number of false-positives and therefore this time series requires closer investigation. A snippet from Rosh Hayin 8 time series (corresponds to October 11^th^, 2008–August 12^th^, 2009) that had several false positives is featured in Fig. 4.

According to the classification rules of this research, this window is considered non-contaminated because no bacteria count in the window exceeded 10 cfu/100 ml. However, at index 2938 the bacteria count was 9 cfu/100 ml, which is on the border of the threshold. The algorithm correctly identified that there was bacteria growth and threw an alarm. Therefore, on closer investigation, these false positives are not actually false—it is only because of the somewhat arbitrary decision to choose 10 cfu/100 ml as the threshold that these were considered false positives.

Evaluating Table 5, there are two main areas of difference between the SVM and the MVE results. On average, MVE predicted events earlier than SVM (Note: there are only 6 events in the Rosh Hayin-8 time series, so each missed event represents 17% of the total). The second difference is that MVE threw fewer false positive than SVM. The MVE algorithm has no false positives compared to the SVM algorithm. These differences can possibly be explained by the nature of the two algorithms. SVM searches for a hyperplane. If such a hyperplane cannot be found SVM projects the problem using a kernel such as polynomials, splines, and radial basis functions^19^. However, these kernel functions are not guaranteed to fit the sample points distribution in the samples domain. MVE, on the other hand is applied on the samples domain and do not need any transform that may or may not fit.

To conclude, spectral methods can be used as predictors of bacteria growth. The energy of the bacteria time series can give an indication of bacteria activity in a well, but it is too crude to be used on its own.

Both the SVM and the MVE algorithms were shown to be able to predict bacteria growth in water wells and provide significant warning time before an event. The main factors that affect the algorithm are the bacteria activity in a well, the training window, the amount of historical data and the threshold by which to define an event. Both methods can be continuously trained as the time series progresses and would become more accurate with time.

All three methods are easily programmed and are cost effective as they rely on data that is mandated by the government for water utilities to have. Most importantly, they provide an objective tool for water quality managers to predict when a bacteria outburst is imminent and who can take prophylactic measures to prevent contamination.

This research has provided the basis for an approach for the prediction of bacteria growth in water wells. The ultimate goal would be its implementation by a water utility. For that to happen, the algorithms should be run on a larger sample size, if possible. A combination of energy, SVM, and MVE should be developed to provide a more robust warning system. A cost evaluation should be performed to understand the ramifications of the false positives on the system. In this regard, a scheme could be created whereby a water engineer would only request another microbial test after several warnings from the algorithm, which would mitigate the cost of the false positives.

## Methods

### Data

Fecal coliform count time series were obtained from the Israeli ministry of health for 37 wells around Israel. The time series were acquired, in irregular time intervals, over twelve years from July, 2001 to December, 2013. The Israeli water regulations mandate a count every three months when the well is in use and no bacteria was found. In case of contamination the sampling frequency is increased. When the well is not operational, no measurements are taken. The average time between samples, for wells where the counts were above zero, is 36 days. The average number of samples acquired in these wells throughout the twelve years period is 159 samples per well.

The water sampling and testing were performed according to the standard methods for examining water and wastewater^35^, so external contamination is avoided. Samples were collected in clean, sterile plastic bottles (LP Italiana, Cat. # 292158, or equivalent), with 100 mg/L Na_2_S_2_O_3_ for dechlorination. The samples were delivered at controlled temperature, below 10 °C (50 °F) within 6 hours to the lab. At the same day the water were tested for coliforms counts. Fecal coliforms counts were acquired through filtering 100 ml of the water sample with Merck Millipore EZ product family or equivalent (EZ-Fit^TM^ Manifold, EZ-Pak^TM^ and Microfil^TM^ Cat # MZHAWG101) membrane^36^. The membranes were removed to 50 mm culture dish with mFC medium (BD Cat. # 267720, or equivalent) and were incubated for 24 ± 2 hours at 44.5 ± 0.2 °C (112.1 ± 0.36 °F). Following the incubation various shades of blue colonies which produced by Fecal Coliform on mFC medium were counted.

Before performing the analysis, an initial classification of the time series was completed and each series was put into one of three categories. This classification is important for determining when the methods prescribed by the research are efficacious. The time series are categorized as follows:

1. “Zeroes” - where all the bacteria counts in the time series were zeroes.
2. “Non-action” – where few of the bacteria counts were non-zeroes, but there was not enough non-zeros so one can make accurate predictions, i.e., not enough historical information. Figure 5a depicts Non-action time series acquired in Machne Yisrael (Israel) water well between March 6^th^, 2001 and December 17^th^, 2013. In these 12 years the well was sampled 107 times, with an average of 44 days between samples and a maximum count of 60 CFU/100 ml. The threshold for bacterial contamination (according to the Israeli standards) is 10 cfu/100 ml, which is marked by a horizontal red line. It is clear from the time series in Fig. 5a that something occurred that caused the sudden bacterial outburst at lot 3571. However, as all the preceding indices are zero, it is impossible to predict said outburst. This is significant as the methods that were employed in this research utilize past information to predict future outcomes. If there is not enough past information, then such a prediction is impossible. The essential difference between the “action” and “non-action” time series is whether there is enough data to accurately predict future outbursts.

**Figure 5:**
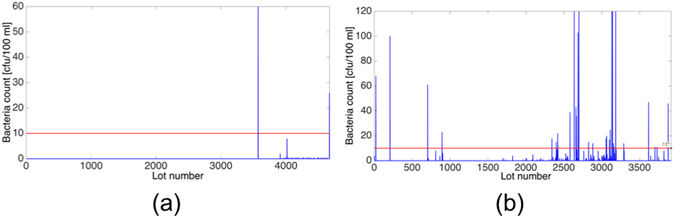
Fecal Coliform Counts. (**a**) Non-action time series acquired in Machane Yisrael well between March 2001 and December 2013. In this time period the well was sampled 107 times with an average time between samples of 44 days; (**b**) Action time series acquired in Rosh Hayin 4 well between April 2001 and January 2011. Throughout this time period Rosh Hayin 4 well 212 counts were performed with an average 19 days between samples.
3. “Action” - where there were a sufficient number of bacteria counts that were non-zeroes, and these phenomena occurred several times throughout the time series. Figure 5b shows an example of an “action” time series that was acquired in Rosh Hayin 4 well between April 4^th^, 2001 and October 26^th^, 2010. The average time between samples was 19 days and the maximum count was 120 CFU/100 ml. It is clear that, as opposed to Figure a, there are several episodes of bacterial growth, which provide enough data for prediction. As in Fig. 5a, the threshold for bacterial contamination, 10 cfu/100 ml, is marked by a horizontal red line.

### Sparseness and Dimension Reduction

A known problem in the signal processing world is the curse of multidimensionality^22–24^. For the purposes of this research, the time series were considered high dimensional spaces. Even though both the original time series and its transform are signals in a 2-d domain (time-space), when they are analyzed using machine learning techniques (which will be elaborated upon below), the time series are cut into a set sequences of predefined length, K. Each sequence is then considered a sample in a K-dimension space. This results in a very high dimensional space. The curse of multi-dimensionality states that as the dimensional space, where the analyses occurs, increases the results of said analysis are worse^37^. This motivates the reduction of the number of dimensions or coefficients in the analysis. To this end, two mechanisms for dimension reduction are presented: signal energy and sparse coding.

#### Signal’s Energy Analysis

Signal’s energy is a characteristic used in signal processing and the Energy (*E*_*s*_) of a continuous time signal x(t) is defined in the signal processing literature as^25^:

2 $${E}_{s}={\int }_{-\infty }^{\infty }{|x(t)|}^{2}dt$$

where *E*_*s*_ is the energy up until time *t*; and *x*(*t*) is the signal’s value at time *t*. The discrete time series equivalent would be the summation of the squared values of the bacteria counts.

The idea behind using energy as an indicator of bacteria activity, is that the higher a count, the higher the energy. Therefore, as bacteria continue to grow, the energy of the signal will also increase. Once the change in energy between two successive counts exceeds a predefined threshold, then one can assume that the pattern of both bacteria and energy growth will continue. An alarm will then be thrown to indicate that there is activity in the well and that said activity should be investigated.

The energy function sums all count information into one scalar representing the time series. A more comprehensive method would be to examine the time-series as a whole. However, the original time series are sparse and, as explained in the introduction, are difficult to process. To this end, this research suggests the compaction of the signal through linear transformation so that an analysis can be conducted on a smaller number of coefficients. The following section explains the rationale behind transforming the time series into a new domain.

#### Sparse Coding

Sparse or compressed coding aims to determine the most condensed representation of a signal, while minimizing distortion in the data. One common method of achieving this is *transform coding*, whereby the signal is transformed to a new basis that allows for sparse representation of the data. A basis Φ is a set of linearly independent vectors that can describe every vector in a given space through linear combinations. Given the following relationship:

3 $$y=\Phi x$$

where *y* is the signal represented in the new space of dimension *k*; *x* is the original signal of dimension *n*; and Φ is the basis operator that preforms the transformation.

If *x* is sparse, then Φ can transform *x* into *y* with only *k* ≪ *n* non-zero coefficients. Meaning, that *x* is represented by *y* through use of just a few linear combinations from basis Φ, and thus *x* can be reconstructed (by preforming the inverse transform) using only the *k* coefficients in *y*. Consequently, most of the energy of the signal is compacted into a relatively small amount of elements^38^.

Candes *et al*.^39^ showed that it is possible to completely reconstruct a signal from partial frequency measurements by solving the following minimization problem:

4 $$({{\boldsymbol{P}}}_{1})\min \,{\Vert x\Vert }_{1}{s}{.tAx}=b$$

This problem is also known as *basis pursuit* ^40^, which calculates the vector with the smallest *l*_1_ norm, and is defined as:

5 $${\Vert x\Vert }_{1}:=\sum _{i}|{x}_{i}|$$

If the original vector is sparse enough that there exists an *x*_0_ that can solve the problem *Ax*_0_ = *b*, then the basis pursuit will calculate it. (*P*_1_) can be expressed as a linear programming problem. The solution to (*P*_1_) results in the original signal as it is the best solution to the above minimization problem. This solution allows for a complete reconstruction of the original signal from only a few of its frequency measurements.

In the above study^39^ *x*_0_ is sought by solving (*P*_1_) when A is the FFT transformation matrix and *b* is a vector that consists of k elements out of the n available transform coefficients. Here Candes’ algorithm was modified so A is the Discrete Cosine Transformation (DCT) matrix. This modification has a great impact on the quality of the results, as the DCT presents better energy compaction than FFT (i.e., DCT presents better capabilities to represent a signal with a fewer number of coefficients)^41^. The definition of the DCT used in this research is^42^:

6 $${X}_{k}=\sum _{n=0}^{N-1}{x}_{n}\,\cos [\frac{\pi }{N}(n+\frac{1}{2})k]\,\,k=0,\ldots ,N-1$$

where *X*_*k*_ is the value of the index (k) in the DCT domain; *x*_*n*_ is the value of the n^th^ index in the original domain (in this case, the time domain); and N is the number of indices in the signal.

If a typical bacteria time series had over 3000 entries, then the original time series could be reconstructed from less than ten percent of the random frequency measurements. This implies that most of the information in a time series is stored in a relatively small number of coefficients. This provides the theoretical underpinnings for the reduction of coefficients in the analysis of the time series.

Once the signal is presented in its compact form, a mechanism to classify signal’s representation as high or low chance for an outburst is required. Two mechanisms for classification are utilized here: (a) Support Vector Machine (SVM)^19^, both are described below.

### Irregular Sampling

One of the issues that this research dealt with was irregular sampling. While the Israeli standards demand that a well be sampled once every three months, the exact date of when to sample is left to the discretion of the water utility and there were instances when sampling did not occur in the prescribed time frame (for example, if a well was shut down for a period of time). Moreover, some wells were sampled more frequently if there was a suspicion of contamination. This irregular sampling hinders the smooth application of statistical procedures^43^. Additionally, there is a need to establish the true time relationship between counts, as two sequential samples taken two years apart do not have the same causal relationship as two samples taken two days apart. For this reason, in this research each sample in a time series was placed in its corresponding date. This resulted in the vast majority of the indices (in this case, time stamps for days) being missing, as on average there are only four samples taken in a given year. Fictitious zeroes were added to replace each missing value. This is not to say that the true value of the counts were zero, as there is no way of knowing this, but rather zeroes were added as a means to conduct the desired analysis.

In order to justify this change to the raw data, all analysis was executed after the DCT was performed on the time series (i.e., after the zeroes were added). It is evident from Equation 6 that if an index in the time domain is zero then it will not have any impact in the DCT domain, as that index does not contribute anything to the summation. Consequently, when the time series are examined after the transformation, the fictitious zeroes do not have any effect on the resulting analysis.

The selection of DCT as the transform was made because the transformation functions are already defined and DCT is relatively easy to compute. Additionally, by using the DCT one can capitalize on the sparseness of a time series. The hypothesis of the research is that the reduction of coefficients will provide for a better signal analyses, and therefore the transform must allow for coefficient reduction without a significant loss of data. DCT has been shown to have relatively good energy compaction^42^ and is therefore a suitable transform.

### Classification

#### Support Vector Machine

Support vector machine (SVM)^19^ is a supervised learning method that is often used for classification. The idea behind SVM is to find, based on training pre-classified data, the optimal hyperplane that achieves the truest separation (minimization problem) while finding the largest margin (maximization problem) between the two classes. SVM is a binary classification technique that uses training data to find the hyperplane and then classifies new data points based on the newly defined separator. The formal definition of SVM is:

7 $$\begin{array}{c}Minimize\,\frac{1}{2}{\Vert w\Vert }^{2}+C\sum _{i=1}^{n}{S}_{i}\\ {s}.{t}:{{y}}_{{i}}({w}^{T}{x}_{i}+b)\ge 1-{S}_{i},\forall i\in \{1,\ldots ,n\}\\ w,b,{S}_{i}\ge 0\end{array}$$

where $w$ is the normal vector to the hyperplane; $C$ is the classifier parameter; ${S}_{i}\,$are the slack variables;$\,n$ is the number of vectors in the training data set;$\,{x}_{i}$ are the training vectors with *m* fetaures;$\,{y}_{i}$ is the classification of for each ${x}_{i}$ and is either 1 or −1; and $b$ is a coefficient that determines the axis intercepts.

The first part of the objective function in equation (7) is the maximization of the margin. The margin is defined as the geometrical distance between the hyperplane and the nearest vectors on both sides of the hyperplane and is expressed as 1/${\Vert w\Vert }^{2}.$ Thus, the minimization of 1/${\Vert w\Vert }^{2}$ is the maximization of ${\Vert w\Vert }^{2}$. The objective function seeks to maximize the margin since as the margin increases the variability between the classes also increases, which results in a cleaner separation. However, the cost of an increasing margin is the increasing probability that a vector will be misclassified. The misclassification error is expressed by the slack variable ${S}_{i},$ which equals zero for a well classified vector, is between 0 and 1 for a vector that lies in the separation region, and is higher than 1 for misclassified vectors. Vectors with a slack value greater than zero are called support vectors. The objective function tries to balance between finding the largest margin while minimizing the misclassification error. The parameter $C$ is used to give more weight to either of those options: a higher value of $C$ causes the penalty for misclassification to increase and therefore reduces the margin area.

#### Minimum Volume Ellipsoid

Minimum volume ellipsoid (MVE)^21^ seeks to find the geometrical minimum ellipsoid that encompasses a data set. As opposed to SVM, where there is training on two distinct classes, in MVE there is training on only one class. The idea is to separate one class from all other types of classes. Once the ellipsoid is found, then any data vector that is located in the ellipsoid is assumed to belong to the newly found class.

The formal definition of the ellipsoid is as follows^44^:

8 $${(x-c)}^{T}\ast A\ast (x-c)=1$$

where $x$ is the data vector; $c$ is the vector that contains the ellipsoid center; and $A$ is the matrix containing the coefficients in the ellipse equations.

To find the minimum ellipsoid, one can formulate the problem as a minimization problem:

9 $$\begin{array}{c}Minimum\,\mathrm{log}|A|\\ s.t.:{({P}_{i}-c)}^{T}\ast A\ast ({P}_{i}-c)\le 1\forall {i}\end{array}$$

where ${P}_{i}$ is the measured vector that the ellipsoid bounds.

The ellipsoid is calculated by the Khachiyan Algorithm^44^. This algorithm finds the minimum ellipsoid by starting from the largest feasible solution that encompasses the training set. The algorithm then, through an iterative process, continually shrinks the ellipsoid until the minimum ellipsoid is found with the acceptable tolerance.

### System Approach

SVM and MVE were used to differentiate between the compact DCT representations of contaminated and non-contaminated wells. A contaminated well was defined as a well where the bacteria count exceeded the allowable threshold of 10 cfu/100 ml, as set by the Israeli standards. Next we present the different stages of the processing pipeline.

#### Pre-Processing

In the pre-processing phase, the bacteria counts are classified as contaminated (not normal) on non-contaminated (normal). To do this, it was assumed that for any bacteria count that exceeded the threshold, the well was contaminated for 90 days prior to the sample. This number was chosen as the window size as it corresponds to the requirement by the standards to sample once in 90 days, which reinforces the notion that 90 days is a significant unit of time for bacterial growth. The necessity to define the well as contaminated prior to the actual contamination is so as to allow the algorithms to learn the phenomena that led to the actual exceeding of the threshold. Thus, if the same phenomena are observed, then the algorithm will throw an alarm before the threshold is exceeded.

The performance of the algorithm relies on the success of the training. One of the parameters that affects the quality of the training phase, is the length of the historical window, which should be long enough so both normal and not normal samples are observed for SVM, and only normal samples for MVE. This compilation is well specific and must be done at the discretion of the researcher, as each well has different growth occurring at different times. In this research, the historical window size was set so a sufficient number of events were recorded and there was enough data to train on. It is clear the more data that you train on, the better the results.

#### Dimension Reduction

For each temporal window, its DCT transform is computed. Pursuant to what was already explained, the reduction of coefficients can yield better results. At this stage, the number of DCT coefficients was 90, as this was the defined window size. For dimension reduction, a small subset of coefficients was arbitrarily chosen. This is done for each window’s transform coefficients. Thus, while the same coefficient locations were used for all the windows, the locations themselves were randomly chosen. These coefficients are later used to classify the contaminated and non-contaminated wells.

#### Building the classifiers

At this stage, Equation 7 and Equation 9 are solved, using the data within the historical window, for obtaining the SVM and the MVE classifiers respectively. The SVM creates two classes, contaminated and non-contaminated. The SVM classifier was calculated using both contaminated and non-contaminated samples. MVE separates one class from all other classes. MVE was used to classify the non-contaminated samples, as water engineers are primarily interested when samples deviate from the norm. Therefore, the MVE was calculated around only the non-contaminated data.

#### Bacterial contamination outbreak prediction

Once the training has been completed, the algorithms can predict bacteria growth. The window of 90 days remains constant, so that as each new count is added, the earliest count in the window is removed. In this fashion, the DCT can be calculated in an iterative fashion^41^, so the transform of each new window’s position can be computed using the transform of the previous location saving computational power and processing time.

Once the transformation is computed, a dimension reduction is executed on the new window. The algorithms then use the classifiers that were previously calculated to determine whether the window is contaminated or not. If the window is classified as contaminated, then an alarm is thrown, which indicates that the algorithm identified a pattern of bacteria growth.
